# Biliary Stricture Following Hepatic Resection

**DOI:** 10.1155/1991/81895

**Published:** 1991

**Authors:** Jeffrey B. Matthews, Philippe Gertsch, Hans U. Baer, Walter P. Schweizer, Leslie H. Blumgart

**Affiliations:** 1 Department of Visceral and Transplantation Surgery University of Berne Berne 3010 Switzerland; 2 Klinik für Viszerale und Transplantationschirurgie Inselspital Bern 3010 Switzerland

## Abstract

Anatomic distortion and displacement of hilar structures due to liver lobe atrophy and hypertrophy occasionally complicates the surgical approach for biliary stricture repair. Benign biliary stricture following hepatic resection deserves special consideration in this regard because the inevitable hypertrophy of the residual liver causes marked rotation and displacement of the hepatic hilum that if not anticipated may render exposure for repair difficult and dangerous. Three patients with biliary stricture after hepatectomy illustrate the influence of hepatic regeneration on attempts at subsequent stricture repair. Following left hepatectomy, hypertrophy of the right and caudate lobes causes an anteromedial rotation and displacement of the portal structures. After right hepatectomy, the rotation is posterolateral, and a thoracoabdominal approach may be necessary for adequate exposure. Radiographs obtained in the standard anteroposterior projection may be deceptive, and lateral views are recommended to aid in operative planning.


*HPB Surgery*, 1991, Vol. 3, pp. 181-191
Reprints available directly from the publisher
Photocopying permitted by license only

© 1991 Harwood Academic Publishers GmbH
Printed in the United Kingdom

### BILIARY STRICTURE FOLLOWING HEPATIC RESECTION

JEFFREY B. MATTHEWS, PHILIPPE GERTSCH, HANS U. BAER, WALTER P. SCHWEIZER and LESLIE H. BLUMGART

*Department of Visceral and Transplantation Surgery, University of Berne, 3010 Berne, Switzerland*

*(Received 8 May 1990, in final form 28 August 1990)*

Anatomic distortion and displacement of hilar structures due to liver lobe atrophy and hypertrophy occasionally complicates the surgical approach for biliary stricture repair. Benign biliary stricture following hepatic resection deserves special consideration in this regard because the inevitable hypertrophy of the residual liver causes marked rotation and displacement of the hepatic hilum that if not anticipated may render exposure for repair difficult and dangerous. Three patients with biliary stricture after hepatectomy illustrate the influence of hepatic regeneration on attempts at subsequent stricture repair. Following left hepatectomy, hypertrophy of the right and caudate lobes causes an anteromedial rotation and displacement of the portal structures. After right hepatectomy, the rotation is posterolateral, and a thoracoabdominal approach may be necessary for adequate exposure. Radiographs obtained in the standard anteroposterior projection may be deceptive, and lateral views are recommended to aid in operative planning.

KEY WORDS: Biliary stricture, hepatic resection, cholangitis, ERCP

### INTRODUCTION

Although injury to the bile ducts during cholecystectomy is the most common cause of benign biliary stricture, iatrogenic stricture may occur after any surgical procedure which involves dissection of the hepatoduodenal ligament or liver hilum, including gastrectomy, pancreaticoduodenectomy, portacaval shunt, and hepatic resection[1,2,3,4,5]. In general, the principles involved in repair of postcholecystectomy stricture apply for these iatrogenic lesions as well, and most cases may be successfully managed by Roux-en-Y hepaticojejunostomy[5,6,7].

Benign stricture developing after prior hepatic resection, however, deserves special consideration. It is well known that hepatic resection results in regeneration of the remaining liver to compensate for the loss of functional parenchymal mass. It is less well appreciated that as a consequence of this process, the structures of the hepatic hilum become markedly rotated and displaced as the liver bulk expands to fill the space left by the resected lobe. Failure to recognize the resultant anatomic distortion renders exposure for stricture repair in this setting particularly difficult and potentially dangerous.

---

Address all correspondence to: Prof. L.H. Blumgart, Klinik für Viszerale und Transplantationschirurgie, Inselspital, 3010 *Bern* Switzerland

Hepatic resection is performed with increasing frequency worldwide, and it is reasonable to expect a parallel increase in the incidence of post-resection biliary stricture. We therefore present three cases of benign stricture following hepatic resection which illustrate the influence of hepatic regeneration on subsequent stricture repair.

### CASE REPORTS

*Patient 1.* A 47 year-old female underwent extended right hepatectomy in April 1983 for a large liver tumor that proved to be focal nodular hyperplasia. Jaundice developed in January 1984, and reoperation with placement of a T-tube in the common bile duct was performed. She had multiple episodes of cholangitis in the subsequent months and was treated with antibiotics. In January 1985, an external biliary fistula through the right lower chest wall developed; by PTC and HIDA scan, this appeared to be a total fistula. ERCP showed a strictured common bile duct. An attempt at operative placement of a U-tube was unsuccessful, and the fistula persisted. In May 1986, an unsuccessful attempt at endoscopic stent placement was made. In October 1986, relaparotomy with the intention of surgical stent placement was attempted, but a duodenal injury was sustained and the common bile duct was not identified. Oesophageal varices were evident on endoscopic examination at this time. She continued on antibiotics until January 1988, when she was referred to this clinic.

She presented with mild jaundice and a total biliary fistula through two sites over her lateral lower chest wall (Figure 1). CT scan revealed prominent left lobe hypertrophy, non-dilated intrahepatic ducts, and splenomegaly. Endoscopy revealed prominent oesophageal varices. There was no ascites. The portal vein and left hepatic artery were patent by angiography. Fistulography demonstrated a connection between the fistula and the intrahepatic bile ducts, but the duodenum was not visualized. Hepatoiodida scan showed delayed uptake and transit of tracer with excretion via the two fistulae. Laparotomy was performed utilizing a right subcostal incision with lateral thoracic extension.

The hepatic hilum was identified at a relatively superficial level along the lateral chest wall. A strictured left hepatic duct in continuity with the biliary fistulae was identified. Roux-en-Y hepaticojejunostomy (70 cm) was performed. A drainage tube was placed into the left hepatic duct and exteriorized through this jejunal loop. A fistula to the duodenum was also encountered; this was repaired via a serosal patch technique over a duodenostomy tube. Portasystemic shunt between the splenic and a large ovarian vein was also performed. The postoperative course was complicated by an upper gastrointestinal hemorrhage from a duodenal ulcer which was managed nonoperatively. Postoperative tubogram and Hepatoiodida scan showed free drainage of bile via the jejunal loop, and the spleno-ovarian shunt remained patent by ultrasound examination. She remains well without jaundice one year later.

*Patient 2.* A 41 year-old female underwent left hepatic lobectomy in 1965 for *Echinococcus alveolaris* infestation. She was treated with Mebendazole until 1986 when she developed Kwashiorkor, and the medication was discontinued. In September 1986 she developed painless jaundice and was referred to this clinic.

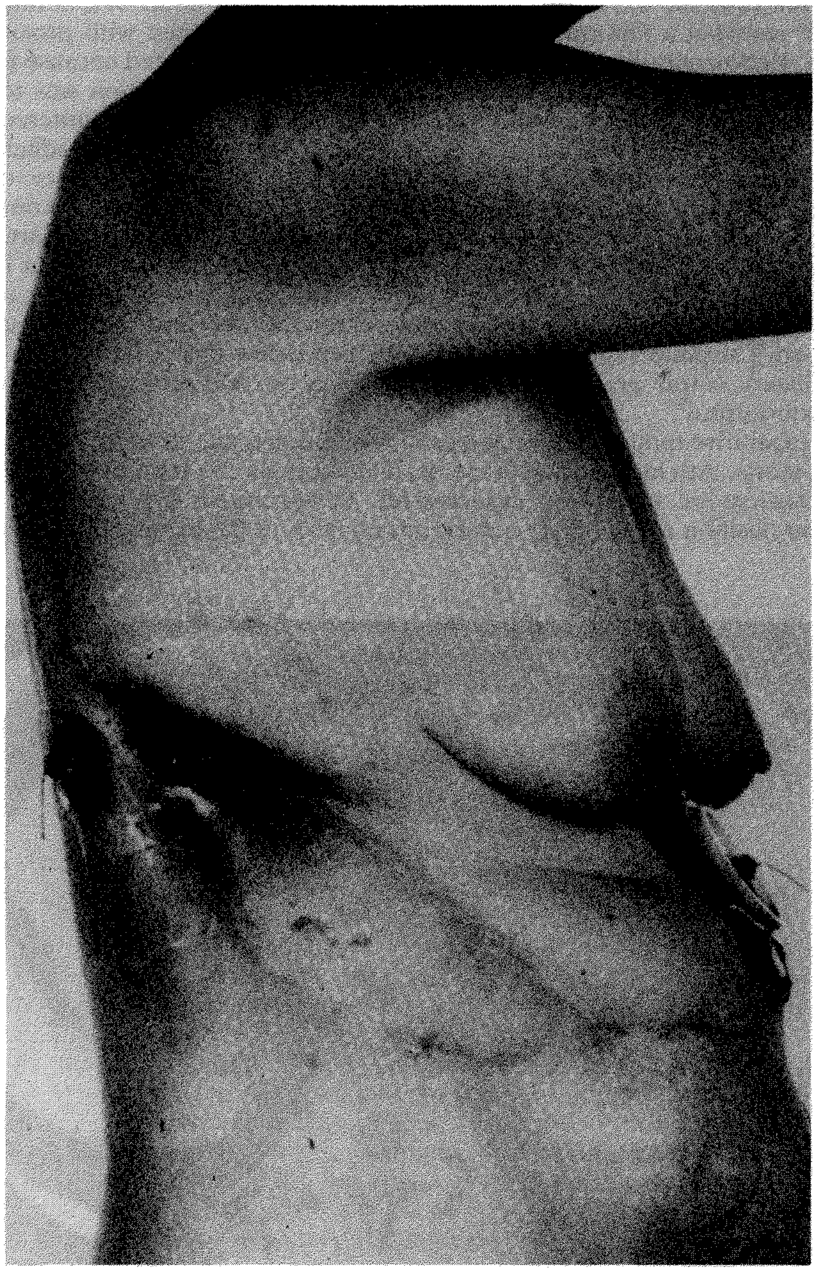

**Figure 1** Multiple external biliary fistulae following extended right hepatectomy (Patient 1). At operation, the hepatic hilus was identified in close proximity to the two fistulae existing over the inferolateral chest wall at the posterior axillary line. Following hepaticojejunostomy, the patient remains well one year later.

At this time she was deeply jaundiced (total bilirubin 164 umol/l, normal < 26). ERCP revealed a filiform stenosis of her right hepatic duct with proximal dilatation. Endoscopic dilatation of this stricture was not attempted because it was felt that the risk of failure with consequent introduction of infection was too great. Laparotomy was therefore performed. The hepatic hilum was encountered directly beneath the subcostal incision near the midline. There were multiple adhesions and areas of prominent chronic inflammation. There was also evidence of portal hypertension (she had undergone multiple sclerotherapy sessions for known oesophageal varices). Injuries to the portal vein and to the right hepatic artery were sustained. The portal vein laceration was repaired with fine suture, but it proved necessary to sacrifice the right hepatic artery. The right hepatic duct was identified in the hilum. Unfortunately, the stricture extended well into the liver substance. Roux-en-Y hepaticojejunosomy was performed below this point and the stricture was dilated. A transanastomotic silicon tube was brought through the jejunal loop and exteriorized[7].

Postoperative tubogram revealed persistent intrahepatic stricture proximal to the anastomosis. Attempts at percutaneous dilatation via the tract of the exteriorized transanastomotic tube were unsuccessful. Postoperative CT scan revealed the anterior position of the biliary-enteric anastomosis at the hepatic hilum (Figure 2).

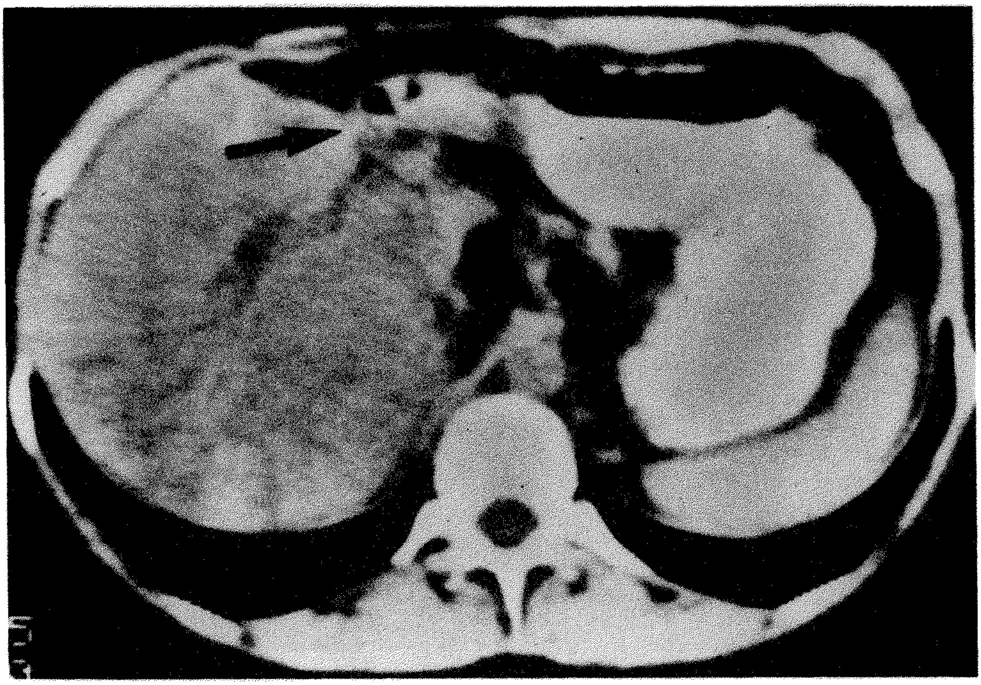

**Figure 2** Postoperative CT scan after hilar hepaticojejunostomy for biliary stricture following left lobectomy (Patient 2). The anastomosis (arrow) has been displaced anteromedially from its usual position as a result of right and caudate lobe hypertrophy.

She recovered and was discharged, but she subsequently has had multiple episodes of cholangitis and developed an hepatic abscess requiring operative drainage. She is currently two years from her operation.

*Patient 3.* A 31 year-old male underwent extended right hepatectomy and atypical partial resection of the left lobe in 1976 for infestation with E. alveolaris. A bile fistula developed 2 years later, and a biliary-enteric anastomosis to a segmental bile duct on the left side was performed. He developed recurrent episodes of cholangitis in 1988 and was referred to this clinic.

Percutaneous transhepatic cholangiogram revealed a tight stricture of the prior biliary-enteric anastomosis and a stenosis of the common hepatic duct at the hilum. The stump of the right hepatic duct, ligated at the prior hepatectomy, could be identified. CT scan (Figure 3), performed immediately after PTC, while contrast material was still present in the biliary tree, revealed the hepatic hilum to be located in a posterior/superior position near the vertebral column. The intrahepatic bile ducts were dilated. Laparotomy was performed via a right subcostal incision with right lateral thoracic extension.

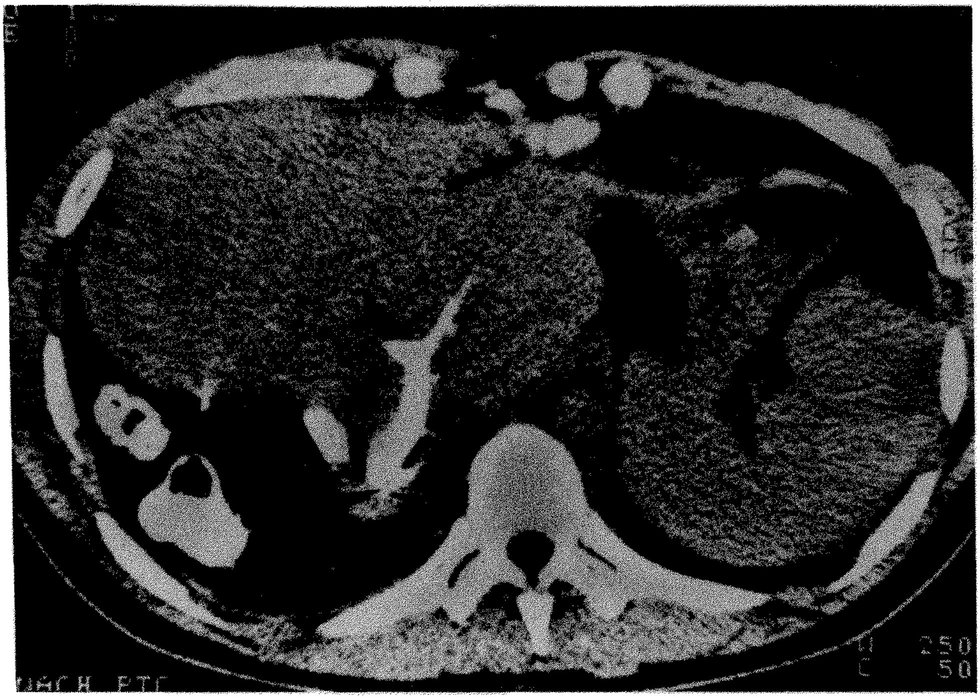

**Figure 3** Preoperative CT scan immediately following PTC in a patient who had undergone prior extended right hepatectomy followed by biliary-enteric anastomosis to a peripheral left segmental duct for biliary stricture with bile fistula (Patient 3). The prior biliary-enteric anastomosis is seen (arrow). Intrabiliary contrast material clearly demonstrates the distorted and displaced position of the hepatic hilus, which is located posteriorly near the vertebral column (double arrowheads).

There were extensive adhesions but no evidence of portal hypertension. The defunctionalized length of the prior Roux-en-Y segment measured approximately 40 cm. The hepatic hilum was identified in a marked posterior position near the vertebral column, confirming the preoperative interpretation of the CT scan. The prior biliary-enteric anastomosis was left intact, but the entero-enterostomy was dismantled. A new 70 cm Roux-en-Y loop was prepared, and hepaticojejunostomy at the hepatic hilum performed. The prior biliary-enteric segment was anastomosed to the new Roux loop. A silicon tube was placed into the original Roux-en-Y segment for the purpose of postoperative imaging.

The patient recovered uneventfully. Postoperative tubogram clearly demonstrated the posterior position of the new hepaticojejunostomy at the hilum (Figures 4 and 5).

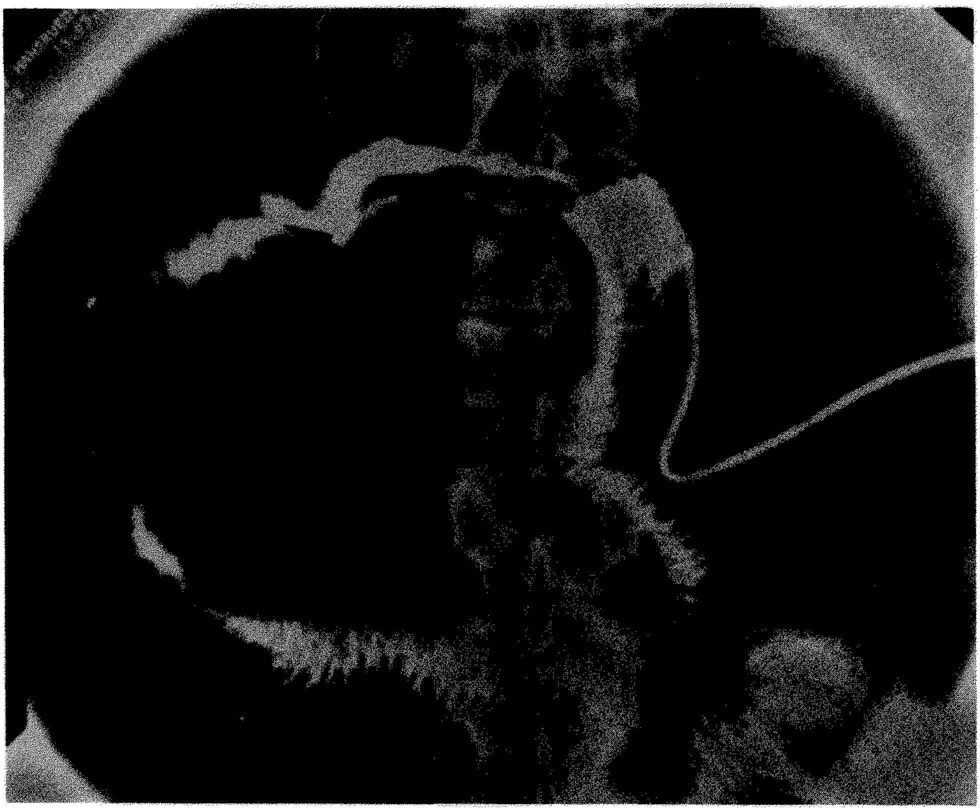

**Figure 4** Postoperative cholangiogram (tubogram) for Patient 3 demonstrates stricture of the prior left segmental hepaticojejunostomy (arrowhead) and a widely patent new biliary-eteric anastomosis performed at the hepatic hilum (arrow). This anteroposterior projection is deceptive, for it does not indicate the marked posterior displacement of the hepatic hilus (see Figure 5).

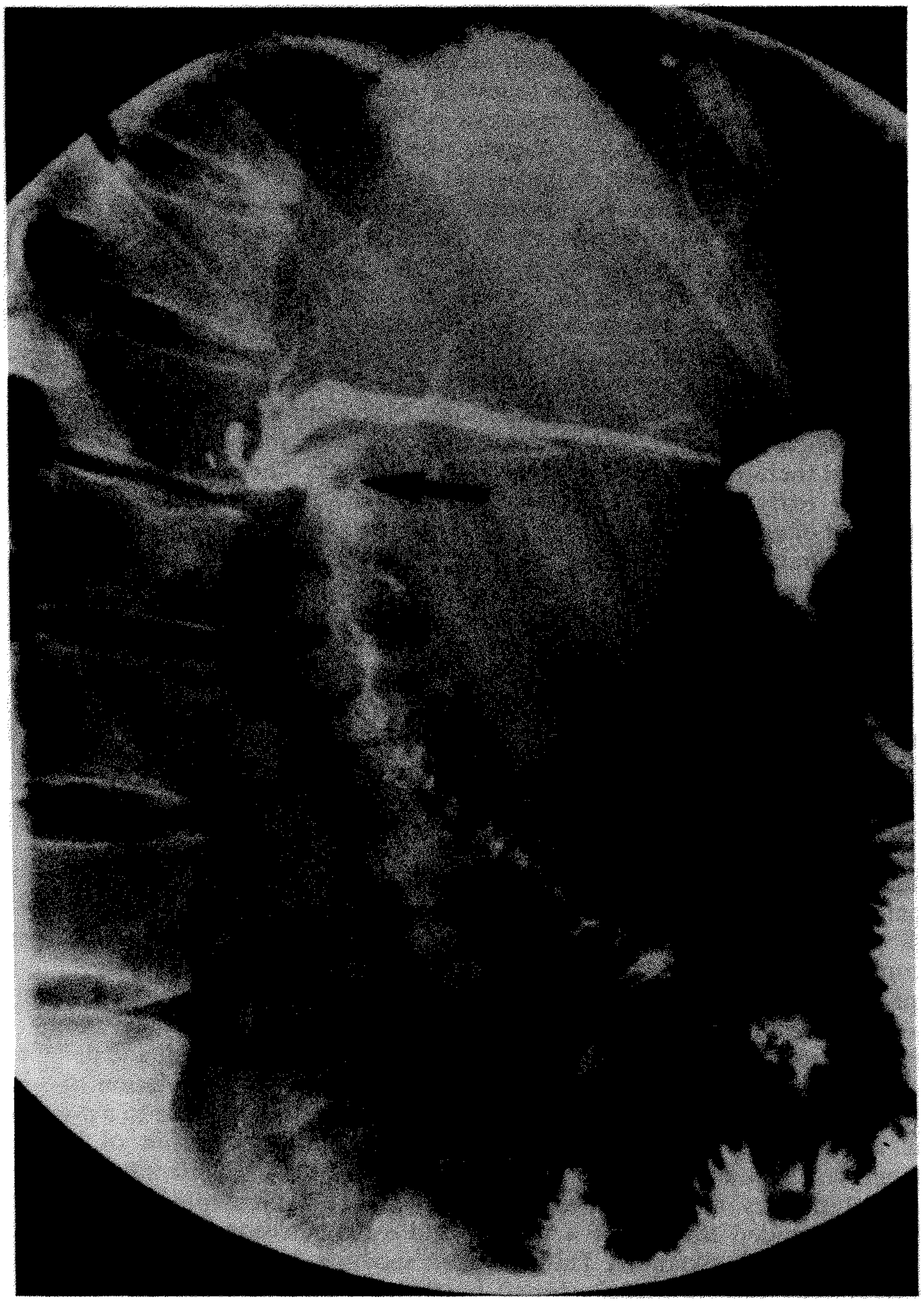

**Figure 5** Lateral projection of postoperative cholangiogram in Patient 3 clearly demonstrates the posterior position of the new hilar hepaticojejunostomy in close proximity to the vertebral column (arrow). The prior anastomosis, with stricture, is anteriorly located (arrowhead).

### DISCUSSION

Benign stricture after cholecystectomy accounts for 90–96% of iatrogenic lesions in most recent series[8,9,10,11]. Stricture following hepatic resection is far less common; only four percent of iatrogenic strictures in the Clevelend Clinic series were the result of operative injury during hepatic resection[11]. The three patients reported here represent nine percent of iatrogenic lesions and five percent of all benign strictures seen over the past 30 months in our unit. Nonetheless, the difficulties inherent to stricture repair in this setting merit special consideration because access to the hepatic hilum is often rather limited and particularly dangerous, critically dependent on the nature of the prior resection. As experience with hepatic resection accumulates worldwide, the hepatobiliary surgeon may encounter this lesion with increasing frequency.

Regeneration of the liver remnant following resection has been recognized for at least a century, and although the responsible biochemical mechanisms have so far eluded complete characterization, this process forms the principle upon which resectional therapy for parenchymal lesions is based[12]. The response is rapid, and significant early growth of the liver remnant is usually readily apparent on ultrasound or computed tomography within days to weeks of hepatic resection. As the liver remnant expands to fill the space left vacant by the resected portion, an inevitable distortion of the hepatoduodenal ligament and hilar structures follows. An analogous distortion of liver anatomy is associated with hepatic lobar atrophy and compensatory contralateral hypertrophy which is encountered in cases of lobar bile duct obstruction or unilateral portal vein thrombosis[13]. The implications of the presence of this so-called "atrophy-hypertrophy complex" regarding subsequent attempts at stricture repair are of great clinical consequence but not widely appreciated[14]. The distortion of liver anatomy following hepatic resection bears an even greater influence on the surgical approach to stricture repair in this setting. Following left hepatectomy, compensatory regeneration of the right hepatic lobe causes rotation of the portal triad toward the midline, and hypertrophy of the caudate lobe causes an anterior displacement of the hepatoduodenal ligament. The hepatic hilum, therefore, may be encountered at a surprisingly superficial location with the portal vein rotated anterolateral to the common bile duct where it thus becomes more vulnerable to operative injury. Because the hepatic hilum is displaced in an *anteromedial* direction, a standard subcostal incision with midline extension should suffice in such cases.

Prior resection of the right hepatic lobe presents the more difficult and dangerous situation for stricture repair. In contrast to the anteromedial rotation observed following left hepatectomy, resection of the right hepatic lobe leads to a *posterolateral* and upward displacement of the hepatic hilum as a result of left lobe regeneration. An analogous posterolateral rotation of the hepatic hilum has been observed in cases of right lobe atrophy and left lobe hypertrophy[13], and Bismuth has recommended the use of a thoraco-abdominal incision via the bed of the seventh rib for this situation[3]. This posterior, superior, and lateral displacement of the hilar structures is well-illustrated by the patients reported here; Patient 1 presented with external biliary fistulae over the lateral chest wall which directly communicated with the hilar ducts. The hilum was located fairly superficially over the inferior chest wall at the axillary line. In Patient 3, the displacement was even more apparent; the hilar structures were located posteriorly in close proximity to the vertebral column. It should be noted that in the presence of significant left lobe hypertrophy, particularly following right hepatectomy, cholangiograms obtained in the standard antero-posterior projection may be deceptive. Lateral views may demonstrate the posterior displacement of the hilum and may therefore be helpful in preoperative planning.

There are two possible approaches to stricture repair following right hepatectomy. Direct access to the hepatic hilum via an anterior abdominal wall incision will generally be inadequate, and therefore the need for a thoraco-abdominal approach should be anticipated in operative planning. An alternative approach in selected cases consists of a modified Longmire operation with hepaticojejunostomy performed to the segment II and/or III sectoral bile ducts. This method has been reported previously[15] and has the advantage of avoiding direct exposure of the hilar structures. However, vascular control with the Longmire procedure tends to be imprecise, and excessive hemorrhage may occur. Furthermore, there may be a higher rate of restricture because of the use of small segmental bile ducts for biliary-enteric anastomosis[16]. The hilar approach is therefore preferable for stricture repair following right hepatic resection.

Hepatic resection is generally a safe procedure in experienced hands, and biliary stricture is no doubt a rare complication. Excessive devascularization of hilar structures may lead to late stricture formation[17] and therefore meticulous, gentle technique combined with a thorough knowledge of hepatobiliary anatomy is essential. Large or bulky tumors encroaching upon the hepatic hilus or lesions associated with a prominent inflammatory or fibrotic reaction (alveolar echinococcosis, e.g.) may render dissection in the hilar region difficult and therefore predispose to bile duct injury. Biliary stricture following hepatic resection poses a particularly challenging problem to the hepatobiliary surgeon, and recognition of the important anatomic consequences of hepatic regeneration is a critical component to successful surgical repair.

### SUMMARY

Three patients with benign biliary stricture following hepatic resection illustrate the influence of hepatic regeneration on subsequent attempts at stricture repair. Following left hepatic resections, hypertrophy of the remaining right and caudate lobes leads to an anteromedial displacement and rotation of the hilar structures in the hepatoduodenal ligament. The more difficult and dangerous situation follows right hepatectomy, after which the hilum is rotated and displaced in a posterolateral direction. A thoraco-abdominal approach will generally be necessary to gain access to the hilar structures in this situation. Cholangiograms obtained in the standard anteroposterior axis may be deceptive, and lateral views are recommended to demonstrate the position of the hepatic hilus. Stricture following hepatectomy is a rare lesion but may be encountered with increasing frequency as experience with hepatic resection accumulates worldwide.

### *References*

1. Florence, M.G., Hart, M.J. and White, T.T. (1981) Ampullary disconnection during the course of biliary and duodenal surgery. *Am. J. Surg.* **142**, 100–105

2. Carpenter, C. and Crandell, W.B. (1958) Common bile duct and major pancreatic injuries during operation on the stomach: a report of three cases. *Ann. Surg.* **148**, 66–72
3. Bismuth, H. and Lazorthes, F. (1981) Les traumatismes operatoires de la voie biliaire principale, Volume 1. Paris: Mason ed.
4. Chadwick, S.J.D. and Dudley, H.A.F. (1984) Stenosis at the choledochojejunostomy anastomosis following pancreatico duodenectomy when the common bile duct is of normal caliber. *J. Roy. Coll. Surg. Eng.* **66**, 319–320
5. Bismuth, H. (1983) Postoperative strictures of the bile duct. In The Biliary Tract, edited by L.H. Blumgart, pp. 209–218. Edinburgh: Churchill Livingstone
6. Blumgart, L.H., Kelley, C.J. and Benjamin, I.S. (1983) Benign bile duct stricture following cholecystectomy: critical factors in management. *Br. J. Surg.* **71**, 836–843
7. Blumgart, L.H. (1988) Benign biliary strictures. In Surgery of the Liver and Biliary Tract, edited by L.H. Blumgart, pp. 721–752. Edinburgh: Churchill Livingstone
8. Way, L.W. and Dunphy, J.E. (1972) Biliary stricture. *Am. J. Surg.* **124**, 287–295
9. Mathisen, O., Bergan, A. and Flatmark, A. (1987) Iatrogenic bile duct injuries. *World J. Surg.* **11**, 392–397
10. Castrini, G. and Pappalardo, G. (1981) Iatrogenic strictures of the bile ducts: our experience with 66 cases. *World J. Surg.* **5**, 753–758
11. Gemest, J.F., Nanos, E.,Grundfest-Broniatowski, S., Vogt, D. and Hermann, R.E. (1986) Benign biliary strictures: an analytic review (1970 to 1984). *Surgery* **99**, 409–413
12. Weinbren, K. and Hadjis, N. (1988) Compensatory hyperplasia of the liver. In Surgery of the Liver and Biliary Tract, edited by L.H. Blumgart, pp. 49–60. Edinburgh: Churchill-Livingstone
13. Hadjis, N.S., Hemingway, A., Carr, D. and Blumgart, L.H. (1986) Liver lobe disparity consequent upon atrophy. *J. Hepatol.* **3**, 285–293
14. Czerniak, A., Soreide, O. and Gibson, R.N. *et al* (1986) Liver atrophy complicating benign bile duct strictures: surgical and interventional radiologic approaches. *Am. J. Surg.* **152**, 294–300
15. Johnson, A.G., Murray-Lyon, I.M. and Blumgart, L.H. (1979) Stricture of common hepatic duct after right hepatic lobectomy treated by Longmire's operation. *J. Roy. Soc. Med.* **72**, 136–139
16. Blumgart, L.H. and Thompson, J.N. (1987) The management of benign strictures of the bile duct. *Curr. Probl. Surg.* **24**, 1–66
17. Terblanche, J., Allison, H.F. and Northover, J.M.A. (1983) An ischemic basis for biliary strictures. *Surgery* **94**, 52–57

*(Accepted by S. Bengmark 28 August 1990)*

### INVITED COMMENTARY

This report of benign strictures of the bile duct which occur or follow a major hepatic resection emphasizes a problem which many hepatobiliary surgeons will encounter during the next few years. All of us who treat liver and biliary problems are beginning to encounter these injuries with increasing frequency. The authors have reviewed their experience in three patients presented and have provided guidelines for surgeons who must repair these problems.

In our experience at the Cleveland Clinic, we have encountered this problem on several occasions. I agree with the approach of the authors. One additional help, in my experience, is to have in place a large transhepatic catheter, placed preoperatively, passed down to the hilar region, so that the surgeon at reoperation can be guided not only by the preoperative percutaneous transhepatic cholangiogram, but also can, by palpation, identify the presence of the catheter as he dissects down to the dilated bile duct. The presence of this catheter has helped us to find a bile duct in two recent patients explored. Additionally, I would caution surgeons who must re-explore patients with a bile duct stricture after a previous hepatic resection, to not re-explore too soon after the previous surgery, since re-exploration at this time can be especially difficult. With a percutaneous transhepatic tube in place, these patients can be carried along with good liver function until the previous area of dissection has "matured", when re-exploration might be undertaken with less difficulty.

Robert E. Hermann
Department of General Surgery
The Cleveland Clinic Foundation
One Clinic Center
9500 Euclid Avenue
CLEVELAND, Ohio 44195–5043
USA

